# Designing body parts

**DOI:** 10.4103/0970-0358.59269

**Published:** 2009

**Authors:** Surajit Bhattacharya

**Affiliations:** IJPS, Sr. Consultant Plastic Surgery, Sahara Hospital, Lucknow, India. E-mail: surajitbh@yahoo.co.in

In the recently concluded IPRAS meeting at New Delhi, while everyone clamoured to hear the masters speak in the main hall, most delegates missed a session in the remote Hall ‘D’. To my mind, that easily was the best session of the meeting. Any guesses what the session was on? It was on Regenerative Medicine and Tissue Engineering.

Regenerative Medicine is a multidisciplinary science that has evolved in parallel with recent biotechnological advances. It is a grand combination of biomaterials, growth factors and stem cells, to repair failing and injured organs. Material scientists can now fabricate biocompatible scaffolds with a wide range of physical parameters, combining mechanical integrity with high porosity to promote cell infiltration and angiogenesis. Likewise, biochemists can produce highly purified, bioactive cytokines in large quantity, suitable for cell culture and *in vivo* applications. Despite these advances, the availability of stem cells remains a challenge for both scientists and clinicians pursuing regenerative medicine

The objective of majority of reconstructive surgery procedures that we perform today is to repair soft tissue defects that result from traumatic injury, tumour resection, and congenital defects. These defects typically result from the loss of a large volume of skin, adipose tissue, muscle tissue and at times bone. Till date, no ideal filler material, which is successful in all cases, has been developed. Composite free tissue transfer is doing the job rather well, but will it not be better if we could regenerate ‘tissue for tissue’? This is where stem cells step in.

Stem cells have the remarkable potential to develop into many different cell types in the body. Serving as a sort of repair system for the body, they can theoretically divide without limit to replenish other cells as long as the person or animal is alive. When a stem cell divides, each new cell has the potential to either remain a stem cell or transform into another type of cell with a more specialized function, such as a muscle cell, a nerve cell or a red blood cell. There are three classes of stem cells: totipotent, multipotent, and pluripotent. A fertilized egg is considered totipotent, meaning thereby that its potential is total; it gives rise to all the different types of cells in the body. Stem cells that can give rise to a small number of different cell types are generally called multipotent. Pluripotent stem cells can give rise to any type of cell in the body except those needed to develop a foetus. Pluripotent stem cells are isolated from human embryos that are a few days old. Cells from these embryos can be used to create pluripotent stem cell “lines” — cell cultures that can be grown indefinitely in the laboratory.

Once a stem cell line is established from a cell in the body, it is essentially immortal, no matter how it was derived. The cell line can be grown in the laboratory indefinitely and cells may be frozen for storage or distribution to other researchers. Stem cell lines grown in the lab provide scientists with the opportunity to “engineer” them for use in transplantation or treatment of disease. Thus, the keratinocye cell line can be used to treat extensive burns and pancreatic cell line can be used to treat diabetes! Stem cell therapy is in clinical and therapeutic trial stages in radiation necrosis, breast reconstruction, breast augmentation, facial aesthetic surgery, tracheal repair, calvarial repair, heart disease, Crohn's disease, and periodontal diseases. It is in pre-clinical trial stages in pulmonary diseases, hepatitis, kidney diseases, Parkinson's disease, urinary incontinence, spinal cord injury, tendon injury, diabetes, stroke, inter vertebral disc renewal, cornea repair, vocal cord repair and muscle repair.

However, research no stem cells derived from foetal tissue is subject to a lot of moral, ethical and legal controversies. The emerging field of regenerative medicine will require a reliable source of stem cells in addition to biomaterial scaffolds and cytokine growth factors. Adipocyte derived stem cells are the current toast of scientists as there is a much larger concentration of stem cells and precursor cells in fat when compared to bone marrow, which is the other well researched source. Adipose tissue represents an abundant and accessible source of adult stem cells with the ability to differentiate along multiple lineage pathways.

Before scientists use any type of tissue, organ, or cell for transplantation, they must overcome attempts at rejection by the immune system. Harvesting autologous fat cells by liposuction, and utilizing them as the source material is what researchers now intend to do. Autologous fat grafting, as practised by aesthetic surgeons, may well be the first regenerative surgery; and the use of adipocytes may not just be as mere biological fillers! Then again, in the future, scientists may be able to modify human stem cell lines in the laboratory by using gene therapy or other miscellaneous techniques to overcome this immune rejection. Scientists might also be able to replace damaged genes or substitute new genes in stem cell lines in order to give them characteristics that can ultimately treat diseases.

So then, how rosy is the path into the future?

Not very! Recent studies have demonstrated, human adipose stem cells, after prolonged culture *in vitro*, are capable of forming tumours in immunodeficient mice. Consequently, long-term experiments examining the safety of adipose stem cell transplantation in appropriate animal models will be required. If and when adipose stem cells are approved for clinical use, physicians and health care professionals will need to be educated regarding their unique properties and correct application. Law makers and the public will have to be taken into confidence. The way embryonal stem cell research was ‘am-Bushed’ should never be forgotten. Blanket bans on technology are almost always mistakes. As a corollary, should we not then ban fertilizers because they can be used to make devastating bombs. Does the promise of a new technology outweigh the risk that it could be misused? The challenges are too important to address in the climate of fear and ignorance, or to be distorted by greed or vain glory of renegade scientists with an agenda to become God! Our fear and ignorance have come in the way of progress for too long.

It is time we clinicians take serious notice of these newer frontiers of research. As Prof. Tom Biggs, the Editor in Chief of Aesthetic Plastic Surgery, quite rightly says ‘This is the decade of fat’! Let us understand the potential of the adipocyte, which we discard in liters following liposuction. Let us collaborate with the basic researchers and reap the maximum bonus from stem cell research and gene therapy. The choice stares at us - either we control the gene technology today or be prepared to be redesigned by it tomorrow.

